# Supplementary material for: During haptic communication, the central nervous system compensates distinctly for delay and noise
Source: PLoS Comput Biol. 2024 Nov 6;20(11):e1012037. doi: 10.1371/journal.pcbi.1012037 (PMC11573204; doi:10.1371/journal.pcbi.1012037)
Supplement: S1 Text — (DOCX) [file pcbi.1012037.s001.docx]

**Questionnaire Items:**

The full list of the questions of the questionnaire used in the study after each experimental block, where questions (1-12) had response options {1/5: strongly dis/agree}:

1. I was controlling the movement I saw.
2. I was the one who produced the movement I saw.
3. It seemed like I felt haptic forces.
4. It seemed like I felt haptic perturbation.
5. It seemed like I felt haptic interaction with an agent.
6. It seemed like I felt haptic noise.
7. It seemed like I felt haptic feedback.
8. It seemed like I felt haptic assistance.
9. It seemed like I felt haptic resistance.
10. It seemed like I felt haptic interaction with a delay.
11. It seemed like I felt that my hand was heavier.
12. It seemed like I felt no haptic feedback of any kind.
13. Was the interaction predictable? {1/5: un/predictable}
14. Was the interaction natural? {1/5: natural/artificial}
15. Was the interaction disturbing or helpful? {1/5 disturbing/helpful}
16. Was the interaction useful? {1/5 useful/harmful}
