# Supplementary material for: During haptic communication, the central nervous system compensates distinctly for delay and noise
Source: PLoS Comput Biol. 2024 Nov 6;20(11):e1012037. doi: 10.1371/journal.pcbi.1012037 (PMC11573204; doi:10.1371/journal.pcbi.1012037)
Supplement: Attachment — Submitted filename: Reviewer_Comments_PCOMPBIOL_D_24_00534.pdf [file pcbi.1012037.s002.pdf]

During haptic communication, the central nervous system compensates distinctly for delay and noise

PCOMPBIOL-D-24-00534

Reviewers' feedback and our response

September 22, 2024

We thank the reviewers and the editor for their work and insightful comments. Please find below how we have addressed these comments point-by-point, with our response in blue color. Within the revised manuscript, text in blue corresponds to changes that have been made as described below.

Response to the Editor

Editor's comments: The reviewers were generally positive about the paper, but did raise some substantial concerns about the modeling for the delay condition. There is a substantial literature on adaptation to delayed feedback which has has argued against the simple delay prediction model put forward here. This literature is largely overlooked, however. In light of the reviews (below this email), we would like to invite the resubmission of a significantly-revised version that takes into account the reviewers' comments and in particular addresses the issues raised by the reviewers surrounding the modeling of behavior in the delay condition.

Our response: We thank you for your work, the feedback and for inviting us to resubmit our paper. Based on your and the reviewers' feedback, we have revised the manuscript particularly to:

- Discuss the relevant literature on adaptation to visual and force delays (lines 297-330).
- Expand on the meaning and expectation of each metric chosen (lines 107-111, 131-132, 148-151 and 154-159).
- Provide additional details as to how the simulation was conducted (lines 205-211, 212-215 and 416-418).
- Add additional statistical analysis in the simulation results that observe differences between the candidate simulation models (lines 242-252).

We have also thoroughly revised the manuscript to clarify sources of confusion identified by the reviewers.

Reviewer 1

The authors present an interesting study of how human actors might account for noise and delay when interacting haptically with humans or robotic assistants to perform joint action tasks. I find the experiments and analysis to be well done and modelling of human behaviour to be convincing and innovative.

1.1: Lines 141 and 154: It is indeed interesting that adding the RP without perturbation both increased the smoothness and decreased the correlation delay. Indeed, I have a question about the comparison between solo and with RP but without added noise or delay. This may have been covered in previous publications, but I have not read them recently and in the interest of time (my review is late) I will pose my questions here. At line 276 it is stated "the information coming from the haptic connection is used to infer the partner's target, which is then combined with their own target in a stochastically optimal manner." In the experiments reported here, what is the RP's "own target"? Was it an accurate version of the actual target? This would mean that the RP is a priori assisting the human subject and the human can infer from instructions or deduce that the haptic information should be helpful and use that information to modulate their own muscle activation's to control the wrist. Or is the RP only moving based on the "inferred" target of its human partner, without independent information about the target's trajectory?

The robot control tracks the target based on its sensory information as well as using noisy information from the haptic channel (where it infers the partner's position information from the interaction force). This yields a human-like interaction (as described in references [4,5] of the revised manuscript). Here, the robot was set to have a skill level consistent with their human user and perform a strategy consistent with what has been previously observed in human-human haptic communication (see [4,5]). This meant that the robot's target measurement had noise added to it that was commensurate with the noise associated to the participant's hand-eye coordination. This equivalence of skill-level means that while the human may be able to infer the relevance of the haptic information, the robot does not have a clearly better performance for the human to exploit.

Here, we note that the amount of noise added to the robot's estimation is determined through the approach used in [5], where a pre-defined mapping has been computed between human RMS error values and the standard deviation of noise that would produce that same RMS error in tracking. The Results (lines 205-211) and Methods (lines 363-365, 367-369 and 397-402) have been revised to clarify how the algorithm works.

1.2: In either case, how do the authors account for the stabilizing effect of the damping in the haptic connector to the RP on the smoothness of the hand trajectory? In other words, what would happen if the RP did not assist the human (position gain set to zero and $\dot{q}_r$ set to zero with the same velocity gain in Eq. 2)? Might this also reduce the tracking error compared to the solo condition, where, from my understanding, no haptic torques are being applied to the participant's wrist? Could the action of the RP in fact be more through a mechanism of impedance matching rather than active assistance and haptic communication per se?

It was indeed a possibility that the improved performance identified during haptic communication studies could have come only from the mechanics of the interaction and not required the exchange of information. Such a model was in fact tested in [4] and compared to the model that has been used in this paper. The findings of [4] were that mechanical interaction alone could not describe the behaviours observed in human-human studies, exhibiting both qualitative differences and a large error relative to the experimental data. This paper has therefore used the haptic communication model which was better able to reproduce human-human behaviour both in simulation and when the model was implemented on a robot partner.

1.3: I find it also interesting that adding noise can elicit the perception of delay. Is this conditioned by the fact that the subjects were told that they would interact with a robot or a human? Was it stated or implied that the robot or human would be acting toward the same goal?

The original phrasing of the experimental methods was misleading and incorrectly suggested that participants were directly told that they would interact with a robot or a human. The exact instruction provided was that participants "may perceive some haptic effects on their wrist". Therefore, while it could be indirectly inferred from their participation with a partner and the use of a haptic device that they were interacting with a robot or a human, it was unlikely that they would realise that the robot or human might be acting towards the same goal.

The Experimental setup and protocol text has been revised to clarify the exact information given to the participants (lines 350-351).

We do agree that it is interesting that adding noise/delay could in some cases elicit the perception of the other modality. This might be a result of the participants being given the option in the questionnaire and making a guess of what they are experiencing based on limited options.

1.4: Aside from this methodological question, I wonder if the author's could consider other studies of haptic perception in face of delays (e.g. Pressman, A., Karniel, A., and Mussa-Ivaldi, F. A. (2006, February). Perception of delayed stiffness. In The First IEEE/RAS-EMBS International Conference on Biomedical Robotics and Biomechatronics, 2006. BioRob 2006. (pp. 905-910). IEEE.)

The manuscript has been updated to include additional works on haptic perception, as well as compensation, in response to delays, within the Discussion section (lines 297-330). This includes the proposed work as well as a subset of the works proposed in Reviewer Comment 2.1.

1.5: And in view of the sharp change in predicted behavior for larger delays (Fig. 6D), I wonder if the author's have considered the so-called binding problem in face of delays? Might the subjects consider small delays as noise when the delay is small and then shift to a compensation as delay strategy when the delay is large, because, hypothetically, with the larger delay the haptic interaction is perceived as a delayed interaction from a separate entity (the RP) rather than a noise perturbation to a single physical interaction?

It is indeed possible that participants switched between considering the perturbation as noise and as delay based on the magnitude of the applied delay. Our choice of simulation models was influenced by the questionnaire and co-contraction results which indicated that participants did not clearly perceive the applied delay as noise, nor did they increase their co-contraction, until the delay was large. These results could indicate that participants might have switched from a delay compensation scheme to a noise compensation scheme with larger delay values. To limit the number of models considered, we chose to model the response to delay as compensation by noise for all delay values if a noise based strategy was considered (where our simulation findings show greater difference between the data and simulation for compensation by noise at large applied delays). A note that the possible strategy for delay compensation may still include some noise compensation, has been added to the revised Discussion of the manuscript (lines 324-330).

1.6: Abstract: Perhaps "Physically connected humans ..." ?

The abstract has been revised to include this change.

1.7: Line 50: Are the experimental finding mentioned here findings from previous studies, or a forward reference to what will be reported here?

The sentence was intended as a forward reference to our experimental findings within the manuscript. The text (line 53) has been revised to clarify this.

1.8: Line 78-79: That subjects more reliably detected delay when there was delay vs. when there was not is clear. It would nevertheless be interesting to know to what extent subjects perceived delay when there was none, i.e. did the responses to the item "It seemed like I felt haptic interaction with a delay." elicit a response that was different from "strongly disagree"?

Thank you for this suggestion. To evaluate this question we conducted a one-sided one-sample Wilcoxon test and compared the zero-delay condition with the "strongly disagree" and "disagree" values to examine whether the median value for the participants' responses were greater. The median was greater than "strongly disagree" ($W = 105, p = 0.8281$), but not different from "disagree" ($W = 22, p < 0.05$) and significantly lower than all other scale values (all $p < 0.05$). We have included this additional information in the revised manuscript (lines 85-86).

1.9: Line 75-81: Could you clearly state which items are used in each analysis, and if you relied on only a single item in each case, or if "perceived noise" (item 6) was somehow conditioned by responses to other items, such as "interaction with an agent" or not?

The analysis of the perception of noise (Item 6) and delay (Item 10) each made use of only a single item. The Results has been revised to clarify which items was used for each analysis.

1.10: Line 85: How is this result "different to the results in the delay group". The sentence confused me because the delay group also perceived delay even for the smallest delay. I suggest: "Participants in the noise group clearly identified the presence of noise ($\chi^2(5) = 66.411$, $p < 0.0001$) as shown in Fig. 3D. In contrast to the perceived noise in the delay group, noise-group participants perceived the presence of noise from the smallest applied noise level ($p < 0.01$ for all pairwise comparisons between 0 mNm and 8-184 mNm noise conditions)."

We agree that the previous text was unclear and have revised the text (lines 92-95 in the updated manuscript) based on the proposed revision.

1.11: Line 90: "none of the conditions were found to be clear different." From each other? Or from the solo or zero-delay conditions? A post hoc test of all pairwise comparisons would be less powerful then a set of planned comparisons between each of the non-zero delays and the zero-delay condition.

This analysis intended to evaluate if the perception of delay was affected by the noise level. As this considered changes over all different noise levels our analysis was performed to evaluate the difference 'from each other'. We have clarified this in the revised manuscript (line 98).

1.12: Fig. 3: I quibble with the axis titles "perceived as ..." and wonder if the delay is orthogonally independent from the noise. Unless the subject knew that there would be only noise or only delay, then the answers to the questionnaire tested only for the perceived presence of noise ("perceived noise") or the perceived presence of delay ('perceived delay"). Whether they perceived delay as noise is a matter of interpretation. Did any subjects perceive delay only as increased noise? The argument that delay is distinguishable from noise also depends on the assumption that no noise is actually added when adding delay. Does introducing delay in fact add noise to the haptic signals received by the subject via the coupling? Could a robotic system take the place of the human and, knowing that the RP will introduce only delay, could the robot identify the delay and produce trajectories that are no noisier than the non-delay system?

The questionnaire items (Item 6 and Item 10) indeed did not ask participants to evaluate their perception of delay or noise exclusively of one another. We have therefore revised Figure 3 as proposed to "perceived noise" and "perceived delay".

1.13: Line 134: The statement is factually correct, but the "in contrast to" perhaps invites an apples-to-oranges comparison. There is no way, in my mind, to calibrate the potential effect of a given level of noise on the tracking performance to the potential effect of a given level of delay on tracking performance. There is no reason to suppose an equivalence between 184 nMn of noise with 540 ms of delay in terms of effect on the tracking error in the absence of co-contraction, so the fact that the co-contraction in the case of delay did not prevent an increase of tracking error is potentially misleading.

The statement was intended to highlight that in the noise group co-contraction increased (for certain noise levels) without there being an increase in tracking error, while in the delay group, the observed increase in co-contraction coincided with an increase in tracking error. The reviewer is correct that no direct comparison can be made between the two groups given that there is no perfect way to calibrate the potential effect of a given level of noise on the tracking performance to the potential effect of a given level of delay on tracking performance. The text has therefore been revised (lines 148-151) to more carefully make the intended observation without implying a comparison between the groups.

1.14: Fig 6: What is the meaning of the different grey lines? Are 6A and 6B simply a different representation of the same results reported in 4A, but with connections between results for individual participants? What is the meaning of the dashed lines?

Figures 6A and 6B were indeed intended to show the individual participant results (with connection for each condition). Here the grey lines indicate an individual participant, the black line denoted the median performance and the dashed lines denoted the interquartile range of participant performance. The caption of Fig 6 has been revised to clarify this intended meaning.

1.15: Why is 6B present, since in the text it is said "To evaluate this, we simulated the experimental scenario (with the same number of trials and blocks) of a participant being connected (by a virtual spring) to a partner with delayed haptic feedback."? Are the authors also simulating the compensation as noise strategy for subjects who received noise perturbations? If so, perhaps an explicit comparison of simulation results in 6E to 6B would also be warranted in the text.

Fig. 6B was included such that readers could compare the trend of behaviour (with the same figure style) between the different simulated delay compensation strategies the experimental response to noise. To clarify this intention we have revised the figure caption and included a direct link to Fig. 6B in the Simulation results section (lines 236-239) which highlights the similar trends of the compensation as noise simulation and noise group experimental data, despite the simulation considering a delay perturbation.

1.16: What is the meaning of the grey lines for the simulation results (6C-6F)? Are these individual simulations tailored to each participant? The way that this was done is not clear to me. And if this is true, does 6C-F represent simulations for both the delay group and the noise group? Or just the delay group?

Similar to Figs. 6A and 6B, the grey lines in Figs. 6C-6E denote individual simulations, where these simulations were created to model the behaviour of only the delay group. As the reviewer correctly supposed, the simulations were indeed tailored to each individual participant. In accordance with the robot partner model first developed in [5], this was achieved by setting the amount of noise in the simulated participant's target measurement such that it produced the same tracking error as the real participant had shown in the solo trajectory tracking. We have revised the figure caption to clarify the meaning of the grey, black and dashed lines. Furthermore, we have revised the Results (lines 212-215) and Methods (lines 416-418) to clarify how the simulated participants were created.

1.17: Lines 184-185: Since the RP is reacting to the haptic information from the human, I wonder if it is the same thing to delay the torque produced by the virtual spring vs. delaying the RP's "own target" estimate.

As the robot's control is proportional to the perceived difference between its target estimate and its estimate of its current position, there is indeed a relationship between delaying the feedback provided by the virtual spring and delaying the robot's "own target" estimate. However, while these two delays might result in similar behaviour (which could be an interesting follow up study) they would not be the same. This is because the virtual spring force acts as a proportional gain on the difference between the participant's and the robot's position. This term differs from the robot's "own target" estimation due to the influence of the robot controller dynamics as well as the robot's use of haptic communication to estimate its partner's target and position.

1.18: Lines 204-210: Have the authors confused 6D and 6F in the text?

Thank you for pointing this typo out. The manuscript has been revised to correctly refer to 6D and 6E as appropriate.

1.19: What is the difference between items 3 and 7? i.e. what is the difference between "haptic forces" and "haptic feedback"? Were there differences in responses to these two items?

In the experiment design, Items 3 was intended to evaluate if participants felt forces at the wrist interface, while

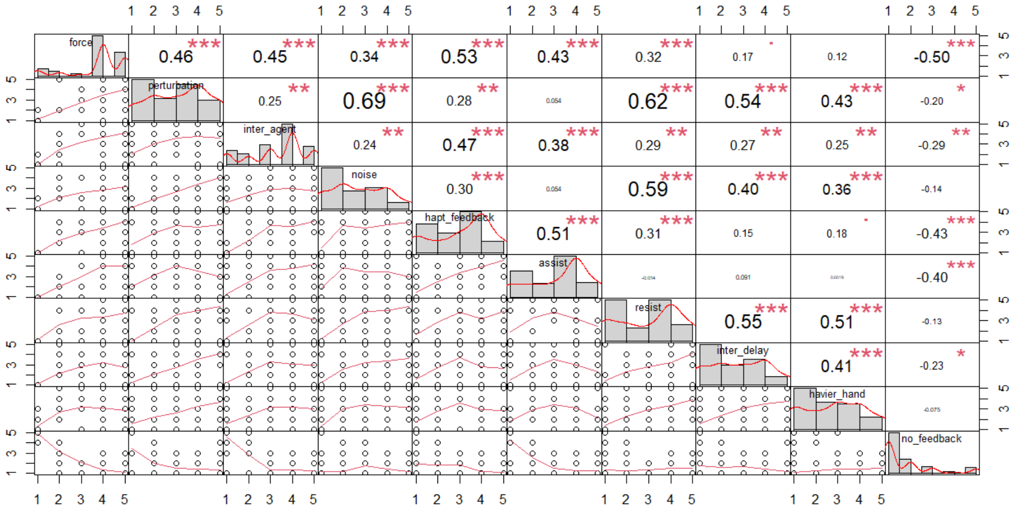

Figure 1: Correlation between questionnaire responses for Items 3-12.

Item 7 was instead intended to evaluate if the participant felt that any force felt was related to the task. The reviewer is though correct that the chosen wording was potentially confusing and as a result there could be some overlap in participant responses. This has been evaluated through a correlation analysis, where there is indeed a high correlation between the answers for the two questions ($\gamma = 0.65$). While this is the case, we preferred to not revise the manuscript for this point, since only Items 3, 6 and 10 were considered in the analysis.

1.20: Were the answers to the questions internally consistent? For instance, if a subject answered non-zero for 8 or 9, did they also answer non-zero for 7 and zero for 12?

Based on this comment we have checked the inter-item consistency across the questionnaire. Here, we first removed Items 1, 2 and 13-15, since these items did not evaluate haptic events. Second, we reversed the scores of Item 12, since this item used an inverted question formulation. The overall average inter-item correlation of the resulting questionnaire, which was calculated as the average of the correlations between all items, was 0.4. This suggests that that there is sufficient consistency between the items and that they still contain enough unique variance (resulting in a lower overall correlation). The average item-total correlation, calculated as the average correlation of the mean response of each person in each condition with the questionnaire items, was 0.67. This implies a good inter-item correlation. The frequency of correlations for both statistics are presented in Figure 1.

Additionally, we used composite reliability and Cronbach's alpha to determine whether the responses were consistent between items. This gave values of 0.86 and 0.87, respectively, which confirms the consistency between the items and, at the same time, does not indicate questions' redundancy.

The Statistical Analysis has been updated to indicate this analysis (lines 471-475).

Reviewer 2

The paper generally addresses an interesting and important problem – haptic collaboration over delayed channels. This problem has many practical implications in today's world and also may shed light on an interesting and unsolved problem of coping with internal delays (however this implication was not mentioned by the authors). The authors nicely extend their previous studies of collaboration between humans and robotic agents in a task of tracking a target, and in the current study they investigate how this interaction is affected by adding noise or by adding a

delay. While the only new experiment is the effect of noise, I do appreciate the analysis of the old and the new study together to make a point and the previous work is correctly acknowledged in the paper. Overall the work is of good quality.

2.1: The authors completely ignore the existing literature about how delays affect movement control and perception. While the particular task of haptic collaboration over a delayed channel was not extensively studied, the effect of delay was studied in several contexts that are related to the task. Except from the failure to put the study in the correct context in terms of prior literature, these prior studies could yield several other possible models for coping with delay except from modeling it as noise. Delay in force feedback was previously shown to change the perception of the mechanical properties of environment (stiffness, mass). Delay in force field adaptation was proposed to be compensated as a combination of current and delayed movement signals, or as a state-based approximation – position, velocity, and acceleration). Delay in visual feedback was proposed to be compensated as a gain, as a mechanical system, or as an altered inertia). All these could result in possible models that can propose explanations for the inaccurate coping with delay beyond noise. While the results of this paper nicely show that delay is not compensated as noise, suggesting that some type of representation or approximation of delay exists, I wander does it make sense to assume such complete lack of representation as an alternative to accurate prediction of time when evidence in favor of alternative ideas exists that are not noise but also not delay representation? Here are a few of the references that are relevant. Note that even though quite a few of these papers are from my group, (not all though), by all means i do not request the authors to cite this entire list. But i would like the author to be aware of these studies and decide how to proceed with this knowledge:

- Pressman et al., 2007, Perception of delayed stiffness
- Nisky et al., 2008, A regression and boundary-crossing based model for the perception of delayed stiffness
- Nisky et al., 2010 A regression and boundary-crossing based model for the perception of delayed stiffness
- DiLuca et al., 2011 Effects of visual–haptic asynchronies and loading–unloading movements on compliance perception
- Leib et al., 2015 The effect of force feedback delay on stiffness perception and grip force modulation during tool-mediated interaction with elastic force fields
- DiLuca Rhodes 2016 Optimal Perceived Timing: Integrating Sensory Information with Dynamically Updated Expectations
- Leib et al., 2017 The mechanical representation of temporal delays
- Avraham et al., 2017 Representing Delayed Force Feedback as a Combination of Current and Delayed States
- Avraham et al., 2017 State-based delay representation and its transfer from a game of pong to reaching and tracking
- Farschian et al., 2018 Energy exchanges at contact events guide sensorimotor integration across intermodal delays
- Avraham et al., 2019 Effects of Visuomotor Delays on the Control of Movement and on Perceptual Localization in the Presence and Absence of Visual Targets
- Van Polanen et al., 2019 Visual delay affects force scaling and weight perception during object lifting in virtual reality

Thank you for highlighting these important previous works which should have been cited in the original manuscript (and has now been done). The intention of our simulation was to validate that the experimental data could not be explained with a 'no compensation' or 'compensation by noise' model such that a model that distinctively incorporated delay compensation was required. Here, we agree that the suggested literature includes a number of viable alternate candidate models for delay compensation including 1) the participant considers the delay as an additional source of impedance in the interaction; 2) they apply a scaling factor onto the current measurement; and 3) they use a weighted average between the current and delayed movement signals. However, since our intent was not to show the exact delay compensation mechanism (for which our experimental data would not be able to separate between), we have in the interest of minimising the number of models presented kept only our original simulation model in the revised manuscript.

We have revised the Introduction (lines 35-36) and Discussion (lines 297-330) sections to clarify that i) our experimental and simulation results together show that the 'no compensation' or 'compensation by noise' models do not appear to match the findings, suggesting that delay is distinctively compensated for (lines 297-300 and 307-311); ii) the literature contains a number of different viable models for distinctive delay compensation including state-based approaches (such as Alternate Compensation Models 1 and 2) and temporal approaches (including the method we present and Alternate Compensation Model 3) (lines 314-318); iii) our simulation results can only show that there is a distinctive delay mechanism and cannot distinguish between the different viable approaches (lines 323-325); and iv) it would be possible to modify the haptic communication model to account for the different models considered previously in the literature. However, this would require additional future studies to obtain data to separate the different models (lines 319-323).

2.2: It is not entirely clear to me how the computational model supports the experimental findings beyond the conclusion that can be reached from the error and co-contraction results? The predictions of the model were consistent generally with the experimental results, but it is difficult to tell if the chosen model indeed explains what happens in the delay case. Indeed the authors are right in their choice of the title stating that the main finding is that delay and noise are not compensated similarly. But this conclusion does not require the computational model. The alternative models I mentioned in the previous point could possibly propose a more compelling explanation.

Through the computational modelling our intent was to validate if the features (both median and extreme behaviours) of the model were consistent with the experimental results. This analysis was done in addition to the experimental analysis as it is possible that i) for this different scenario, participants did no compensation and the results are merely a result of the dynamics of the task being such that the differences were not clear until the delay was sufficiently large; ii) that while participants did not use the co-activation strategy based on the experimental data, they may have engaged in a different compensation by noise approach given that this kind of behaviour has been previously shown to model the differences in haptic communication with different stiffness levels. We agree that our computational modelling cannot find the exact mechanism that is used for delay compensation. However, through the modelling our results suggest that the *no compensation* strategy results in unrealistic extreme case behaviour that is not replicated in the data, while the *compensation as noise* case results in a different trend that would be observed in the data. The purpose of the modelling and the significance of its results has been updated in the revised manuscript (lines 285-296 and 307-309).

2.3: It would help the interpretations a lot if the authors would explain exactly what are their prediction with respect to each of the computed metrics, and mentioned the specific results in the narrative rather than just the statistical results. For me the result that was most difficult to understand due to the lack of predictions and narrative is the results of the delay cross-correlation – did they expect the delay to change? To which value? What the exact value of the delay teaches us? The authors state in the discussion that: "The reduced lag then suggests that this information not only improves the quality of their estimation but also aids them to adapt quicker then what they

normally would" but it is not clear to me why. The other results are a bit more intuitive, but still the way they are presented could be improved – I would like to be able to get an idea about the exact result from the text, and then go to the figure to make sure I agree with the authors based on what I see.

The cross-correlation delay measures the temporal difference between the target and participant trajectory. In the solo condition and for interactions with the robot partner but without delay, due to the trajectory having a random start point it was expected that this value would be small but positive reflecting the participant's need to react to the target motion. When a delay perturbation was added, due to the delayed haptic connection potentially slowing the user down, the cross-correlation delay would be expected to increase for an uncompensated for delay perturbation.

Our results show three main findings:

- The addition of the robot partner reduced the cross correlation delay. This result has not been previously evaluated in interaction studies and could suggest that the additional partner feedback aids by either being quicker to react than the participant, or by aiding their prediction of the trajectory;
- For the delay group there is no clear increase in cross-correlation delay until the high delay values. This suggests that participants are able to use a compensation mechanism that works for small delay values but that breaks down at the high delay magnitudes (although the overall lag was still the same size as that of working in the solo condition);
- The noise group does not show a clear increase in cross-correlation and if anything decreases with increasing cross correlation. This finding suggests that the presence of the noise may also be making the participants more reactive.

These findings have been clarified in the revised Results (lines 163 and 173-174) and Discussion (lines 263-275 and 281-284) sections. Additionally for each metric the Results section has been revised to more clearly state the reasoning and expectation for the individual metric, see lines 107-111, 131-132, 148-151 and 154-159.

2.4: Similarly, the exact interpretation of the simulations are not entirely clear. For example, I could not tell based on figure 6 whether a real difference exists between the predictions of the no-compensation and the compensation as delay, which is confusing and makes the message of the paper less clear.

Thank you for pointing out the previous lack of clarity. The key difference between the no-compensation and the compensation as delay model predictions was in the extreme behaviour (for participants with less skill). In this case the no-compensation model displayed unstable behaviour that was not seen in either the experiments or in the compensation as delay model. We have revised the manuscript (lines 219-223) to clarify this point. However, because this based on the extreme behaviour, we note that the evaluation is qualitative.

We have also revised the Simulation results (lines 242-252) to include a statistical analysis of the difference in between-participant variance for the different simulation models and the delay group experiment results. Details of how this analysis was conducted have also been added to the Methods (lines 484-486).

2.5: The illustration of the different models in Figure 1 is also not clear – why at some point the compensation as delay diverges from the original trajectory. In general, in addition to the general illustration becoming more clear, I would like to see simulated trajectories and experimental trajectories to be able to compare the predictions of the model to the data.

In Figure 1, since our intention was to show a delay compensation strategy, in which the human might use a prediction, we separated the delay prediction (dashed line) from the true trajectory (solid line) such that both elements could be clearly seen. This point has been clarified in the figure caption.

2.6: Minor: they call the connection between the partners an elastic band but I believe a viscoelastic band is more accurate.

The revised manuscript has been changed to use the terms ‘viscoelastic band’ and ‘viscoelasticity’.
